# Low Serum Melatonin Levels Prior to Liver Transplantation in Patients with Hepatocellular Carcinoma are Associated with Lower Survival after Liver Transplantation

**DOI:** 10.3390/ijms20071696

**Published:** 2019-04-05

**Authors:** Leonardo Lorente, Sergio T. Rodriguez, Pablo Sanz, Pedro Abreu-González, Agustín F. González-Rivero, Antonia Pérez-Cejas, Javier Padilla, Dácil Díaz, Antonio González, María M. Martín, Alejandro Jiménez, Purificación Cerro, Julián Portero, Manuel A. Barrera

**Affiliations:** 1 Intensive Care Unit, Hospital Universitario de Canarias. Ofra, s/n, La Laguna-38320, Santa Cruz de Tenerife, Spain; 2 Intensive Care Unit, Hospital Universitario Nuestra Señora Candelaria. Crta Rosario s/n. Santa Cruz Tenerife-38010, Spain; sergiotomasr@hotmail.com (S.T.R.); mar.martinvelasco@gmail.com (M.M.M.); 3 Department of Surgery, Hospital Universitario Nuestra Señora de Candelaria, Crta Rosario s/n, Santa Cruz Tenerife-38010, Spain; sanzpereda@gmail.com (P.S.); javipq@me.com (J.P.); mbargom@yahoo.es (M.A.B.); 4 Department of Phisiology, Faculty of Medicine, University of the La Laguna, Ofra, s/n. La Laguna-38320, Santa Cruz de Tenerife, Spain; pabreu@ull.es; 5 Laboratory Department, Hospital Universitario de Canarias, Ofra, s/n, La Laguna-38320, Santa Cruz de Tenerife, Spain; agonriv@hotmail.com (A.F.G.-R.); aperezcejas@gmail.com (A.P.-C.); 6 Department of Digestive, Hospital Universitario Nuestra Señora de Candelaria, Crta Rosario s/n, Santa Cruz Tenerife-38010, Spain; ddiazbet@gmail.com (D.D.); angonrod@gobiernodecanarias.org (A.G.); 7 Researcher, Research Unit, Hospital Universitario de Canarias, Ofra, s/n, La Laguna-38320, Santa Cruz de Tenerife, Spain; ajimenezsosa@gmail.com; 8 Transplant Unit, Hospital Universitario Nuestra Señora Candelaria, Crta Rosario s/n, Santa Cruz Tenerife-38010, Spain; pcerlop@gmail.com; 9 Department of Radiology, Hospital Universitario Nuestra Señora Candelaria, Crta Rosario s/n, Santa Cruz Tenerife-38010, Spain; julxabpornav@gmail.com

**Keywords:** melatonin, hepatocellular carcinoma, liver transplantation, mortality

## Abstract

Melatonin administration has been associated with different benefits in animals and patients suffering from liver diseases. However, there is no published data about circulating melatonin levels in patients with hepatocellular carcinoma (HCC) who underwent liver transplantation (LT). Thus, the objective of this observational and retrospective study was to determine whether patients with HCC with lower serum melatonin levels prior to LT have a higher risk of one-year mortality after LT. We measured serum levels of melatonin, malondialdehyde (to assess lipid peroxidation), and total antioxidant capacity (to assess antioxidant state) before LT. One-year surviving LT patients (*n* = 129) showed higher serum levels of melatonin (*p* = 0.001) and total antioxidant capacity (*p* = 0.001) and lower serum levels of malondialheyde (*p* = 0.01) than non-surviving LT patients (*n* = 16). Logistic regression analysis showed that high serum melatonin levels prior to LT were associated with lower one-year LT mortality (odds ratio = 0.525; 95% confidence interval (CI) = 0.331–0.834; *p* = 0.006). We found an association between serum levels of melatonin with serum levels of malondialheyde (rho = −0.22; *p* = 0.01) and total antioxidant capacity (rho = 0.21; *p* = 0.01). Thus, the novel findings of our study were the association between high serum melatonin levels prior to LT and survival at first year after LT and the association between serum levels of melatonin with malondialheyde and total antioxidant capacity.

## 1. Introduction

Hepatocellular carcinoma (HCC) is the most frequent primary malignant liver tumor and a frequent cause of cancer-attributable death, and the choice of treatment for some of these patients is liver transplantation (LT) since it removes the tumor and treats the liver insufficiency [1,2,3,4,5,6,7,8].

Melatonin is synthesized mainly with a circadian rhythm (with a low production during the day and high production at night) in the pineal gland and without a circadian rhythm by other organs (gastrointestinal tract, bone marrow, thymus, and lymphocytes) [9]. Melatonin plays an important role in sleep regulation [10]. Besides, melatonin has other effects as an antioxidant, anti-inflammatory, and preserver of mitochondrial function, and its use has been proposed in LT and in HCC [11,12,13,14]. In animal models, the administration of melatonin has been associated with better liver function, lower increases in transaminases, lower oxidation, and higher survival rates after liver ischemia/reperfusion or the injection of hepatoma cells or an inductor agent of HCC [15,16,17,18,19]. In addition, the administration of melatonin has been associated with better liver function and lower increases in transaminases in patients undergoing hepatectomy or with unresectable HCC [20,21,22,23]. However, there is no published data about circulating melatonin levels in patients with and without HCC who have undergone LT. Thus, the objective of this study was to determine whether patients with HCC and lower serum melatonin levels prior to LT have a higher risk of one-year mortality after LT.

## 2. Results

A total of 145 patients were included in the study. We found that one-year surviving LT patients (*n* = 129) in comparison to non-surviving LT patients (*n* = 16) showed younger LT donors (*p* = 0.03), higher serum levels of melatonin (*p* = 0.001), total antioxidant capacity (*p* = 0.001), and lower serum levels of malondialheyde (*p* = 0.01) (Table 1). However, we found no statistically significant differences between survivors and non-survivors in LT age receptor, sex, ABO blood type, serum AFP levels, nodule size, degree of tumor differentiation, multinodular tumor, infiltration, microvascular invasion, macrovascular invasion, portal hypertension, Child-Pugh score, inside Milan criteria prior to and after LT, MELD score, treatment prior to LT, or LT technique (Table 1). The 16 causes of death were the following: nine (56.2%) were sepsis, four (25.0%) were multiple organ failure, two (12.5%) were HCC recurrence, and one (6.2%) was recurrence of hepatitis C virus infection. The patient that died due to recurrence of hepatitis C virus infection remained alive 110 days after LT, and the two patients that died due to HCC recurrence remained alive 85 and 250 days after LT, respectively.

**Figure 1:**
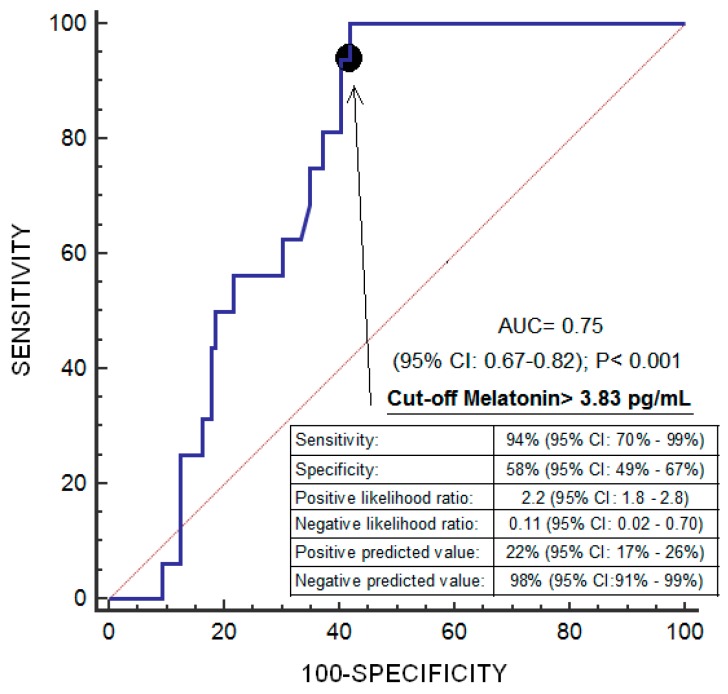
Receiver operation characteristic (ROC) of serum melatonin levels prior to liver transplantation due to hepatocellular carcinoma for the prediction of one-year liver transplantation survival.

**Figure 2:**
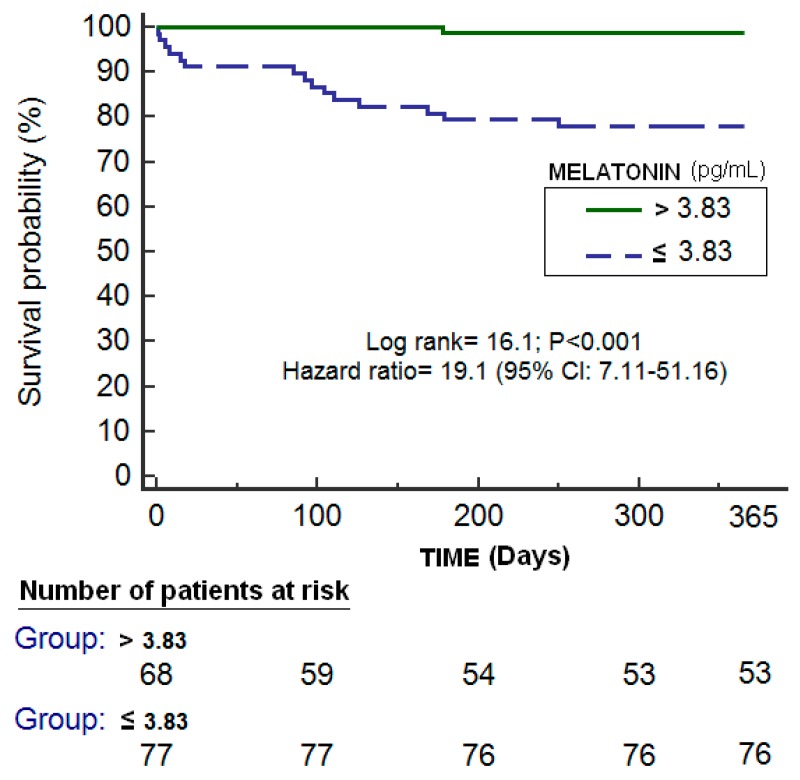
Kaplan–Meier survival analysis using one-year liver transplantation mortality (as dependent variable) and serum melatonin levels prior to liver transplantation lower/higher than 3.83 pg/mL (as independent variable).

Logistic regression analysis showed that high melatonin levels prior to LT were associated with lower one-year LT mortality (odds ratio = 0.525; 95% CI = 0.331–0.834; *p* = 0.006) (Table 2). ROC analysis showed an area under the curve of 75% (95% CI = 67–82%; *p* < 0.001) for the prediction of one-year LT mortality by serum melatonin levels prior to LT (Figure 1). Kaplan–Meier survival analysis showed that patients with serum melatonin levels prior to LT lower than 3.83 pg/mL had a higher one-year LT mortality risk (hazard ratio = 19.1; 95% CI = 7.11–51.16); *p* < 0.001) than patients with higher concentrations (Figure 2). We found an association of serum melatonin levels with serum levels of malondialheyde (rho = −0.22; *p* = 0.01) and total antioxidant capacity (rho = 0.21; *p* = 0.01) but not with serum levels of protein (rho = −0.09; *p* = 0.33), albumin (rho = −0.001; *p* = 0.99), or BMI (rho = −0.14; *p* = 0.10). We found no statistically significant differences in serum melatonin levels between patients with and without ascites prior to LT (*p* = 0.16).

We repeated the statistical analyses excluding patients that died due to recurrence of hepatitis C virus infection or HCC recurrence, and the results were similar to logistic regression, ROC analysis, and the association of serum melatonin levels with serum levels of malondialheyde and total antioxidant capacity.

## 3. Discussion

Previously, the administration of melatonin has been associated with different benefits in animals [15,16,17,18,19] and patients [20,21,22,23] with liver diseases. In rats, the administration of melatonin has been associated with better liver function, lower increases in transaminases, and lower oxidation after liver ischemia/reperfusion [15,16,17]. In a study of rats injected with hepatoma cells, the administration of melatonin was associated with lower tumorigenic activity and higher survival [18]. In another study, where rats received an injection of an inductor of hepatocarcinogenesis, the rats also receiving melatonin showed lower tumor development and lower increases in transaminases and malondialheyde [19]. In a study with human hepatocytes isolated from liver pieces resected from 10 patients undergoing partial hepatectomy, the administration of melatonin was associated with higher cell viability [20]. In one study, 50 patients with a major liver resection were randomized to receive or not receive a preoperative single dose of melatonin administered through the gastric tube after the intubation, and the melatonin treatment group showed lower postoperative serum transaminases concentrations, a shorter intensive care unit stay, and a shorter total hospital stay [21]. In a study of 100 patients with inoperable advanced primary hepatocellular carcinoma and treated with transcatheter arterial chemoembolization (TACE), half of the patients also receiving melatonin showed better liver function and higher survival [22]. In a study of 14 patients with unresectable HCC (due to locally advanced or metastatic HCC) and receiving melatonin, tumour regression was found in five patients (36%), stable disease was found in six (43%), and disease progression was found in three (21%) [23].

However, there is no published data about circulating melatonin levels in patients with HCC who underwent LT. Thus, the association between high serum melatonin levels prior to LT and survival at first year after LT is a new finding of our study. In addition, we are the first to find an association of serum levels of melatonin with serum levels of malondialheyde (a negative association) and total antioxidant capacity (a positive association) prior to LT—and hence an association between melatonin and states of oxidation and antioxidation. It is possible that non-surviving LT patients, compared with surviving patients, retain lower serum levels of melatonin and a lower total antioxidant capacity as well as higher serum levels of malondialheyde during the first year after LT. The anti-inflammatory effects of melatonin reduce proinflammatory cytokines such as interleukin (IL)-6 and tumor necrosis factor-α, and this increases anti-inflammatory cytokines such as IL-10. The antioxidant effects of melatonin act as a scavenger of reactive oxygen species (ROS), which increases antioxidant enzymes such as glutathione peroxidase and glutathione reductase.deoxyribonucleic acid deoxyribonucleic aciddeoxyribonucleic acid deoxyribonucleic aciddeoxyribonucleic acid (DNA). The preserver effects of the mitochondrialdeoxyribonucleic acid function by melatonin reduce ROS and protect the mitochondrialdeoxyribonucleic acid respiratory enzyme complex. We think that it is possible that surviving patients have a lower inflammatory state, a lower oxidative state, and better mitochondrial function than non-survivors prior to LT and during the first year after LT. All these effects of melatonin as an antioxidant, an anti-inflammatory, and a preserver of mithochondrial function might influence LT survival.

Our study has some limitations. First, we have no data about the melatonin and oxidative status in liver tissue. Second, we did not determine serum melatonin levels in healthy subjects or in cirrhotic patients; however, the objective of our study was not to compare serum melatonin levels between HCC patients and other populations but rather to determine whether there is an association between serum melatonin levels and survival after LT. Third, we found that serum melatonin levels in our patients were lower than expected according to kit instructions, which reflects that healthy subjects show a marked circadian rhythm characterized by low levels during daytime (up to 30 pg/mL) and high levels during nighttime (up to 150 pg/mL), and according to findings in cirrhotic patients that showed noctural and diurnal serum melatonin levels around 53 pg/mL [24]. However, the serum melatonin levels that we found in patients with hepatocellular carcinoma prior to liver transplantation were similar to those we found previously in patients with sepsis [25,26], traumatic brain injury [27], and brain infarction [28]. Fourth, blood samples were not obtained exactly at the same time of day for each patient, and it is known that melatonin liberation has a circadian rhythm (with higher values during the night than during the day). However, all patients come from home to hospital for LT, and all blood samples were collected approximately at the same time of the day, between 3 and 6 p.m. In addition, we measured light intensity in the intensive care unit (ICU) at different moments of the day and found few changes throughout the day. We found a light intensity of 2.8 lux in the morning (corresponding to the light period and the greatest ICU activity) and of 0.2 lux at night (corresponding to the dark period and the least ICU activity); thus, the ratio of ICU light intensity between light and dark periods is approximately 14. However, the light intensity outside the hospital varies from 1000 lux in the morning to 0.1 lux at night; thus, the ratio of outdoor hospital light intensity between light and dark periods is approximately 10,000. Fifth, the determination of serum melatonin levels at different moments of day and during the year after LT could help to better determine the melatonin status of patients. Sixth, melatonin production is dependent on tryptophan (an essential amino acid of the diet) [29,30], muscle mass is dependent on nutritional status [31,32,33,34], and LT outcome is dependent on nutritional status [31,32,33,34]; thus, it would have been interesting to determine whether an association between melatonin, tryptophan, nutritional status, muscle mass, and LT outcome exists. However, we have not found statistically significant differences in serum levels of proteins and albumin between surviving and non-surviving patients, and we have not found an association between serum levels of melatonin, proteins, or albumin. Seventh, we have no data about the quality of the organs transplanted.

We think that the findings of our study will not change clinical practice. However, we think that the results of our study could raise interest in research on the role of serum melatonin levels prior to LT for the prediction of survival after LT and the potential use of melatonin administration to reduce the risk of death after LT (which patient, when to start treatment, the dosage, and the duration of treatment). In addition, we think that, although we have found that higher serum melatonin levels have been associated with LT survival, this does not mean that LT at night leads to a better outcome (since melatonin levels are 10 times higher during the night than during the day) but that patients with higher serum melatonin levels at the same time of day would have a greater chance of LT survival.

## 4. Methods and Materials

### 4.1. Design and Patients

We included patients with HCC who underwent LT in the Hospital Universitario Nuestra Señora de Candelaria (Santa Cruz de Tenerife, Spain) between January 1996 to May 2017. All LT donors were brain-dead. This observational and retrospective study was carried out with the approval of Institutional Review Board and with the written informed consent of patients or a member of their family.

### 4.2. Variables

We used one-year LT survival as our end-point study. We recorded sex, ABO blood type, age, Child-Pugh score [35], size nodules, degree of tumor differentiation, infiltration, macrovascular invasion, microvascular invasion, multinodular tumor, portal hypertension (determined clinically or by hepatic venous pressure gradient), model for end-stage liver disease (MELD) score [36] by hepatic function, serum alpha-fetoprotein (AFP) levels, inside Milan criteria [37] prior to and after LT, treatment prior to LT, and the LT technique.

Serum blood samples were obtained approximately 2 h before LT and frozen at −80 °C. We previously determined serum levels of malondialdehyde (to assess lipid peroxidation) [38] and total antioxidant capacity (to assess antioxidant state) [39] in some of these patients. In this work, we determined serum levels of melatonin.

### 4.3. Determination of Serum Levels of Melatonin, Malondialdehyde, and Total Antoioxidant Capacity

Serum levels of melatonin and malondialdehyde were determined in the Physiology Department of the Faculty of Medicine from the University of La Laguna (Tenerife, Spain), and the total antioxidant capacity was determined in the Laboratory Department of Hospital Universitario de Canarias (La Laguna, Tenerife, Spain). We determined melatonin levels with a kit of Immuno Biological Laboratories (IBL Hamburg GmbH, Hamburg, Germany), which had a detection limit of 0.13 pg/mL, an intra-assay coefficient of variation (CV) of 6.4%, and an inter-assay CV of 11.1%. Malondialdehyde determinations were carried out with a thiobarbituric acid-reactive substance method [40], which had a detection limit of 0.079 nmol/mL, an intra-assay CV of 1.82%., and an inter-assay CV of 4.01%. We determined total antioxidant capacity using a kit of Cayman Chemical (Ann Arbor, MI, USA), which had a detection limit of 0.04 nmol/mL, an intra-assay CV of 3.4%, and an inter-assay CV of 3.0%.

### 4.4. Statistical Methods

Categorical variables were reported as frequencies (and percentages) and compared by a chi-square test, and continuous variables were reported as medians (and percentiles 25 and 75) and compared by Mann–Whitney test. The capacity prediction of one-year LT mortality by serum levels of melatonin prior to LT was estimated using the receiver operating characteristic (ROC) curve. We used the Youden J index for the cut-off selection of serum melatonin levels in the Kaplan–Meier one-year LT survival analysis. The possible association between serum melatonin levels prior to LT and one-year after LT was determined by logistic regression analysis, and was reported in odds ratios (and 95% confidence intervals). The association of serum melatonin levels prior to LT with serum levels of malondialheyde and total antioxidant capacity was explored by Spearman’s rank coefficient and Bonferroni’ correction for multiple comparisons. The programs SPSS 17.0 (SPSS Inc., Chicago, IL, USA) and MedCal 15.2.1 (Ostend, Belgium) were used for the statistical analyses, and *p*-values <0.05 were considered statistically significant.

## 5. Conclusions

The novel and more important findings of our study were that there is an association between high serum melatonin levels prior to LT and survival at first year after LT and that there is an association of serum levels of melatonin with malondialheyde and total antioxidant capacity.

## Figures and Tables

**Table 1:** Clinical characteristics of one-year liver transplantation surviving and non-surviving patients.

	1 Year Non-Surviving Patients (*n* = 16)	1 Year Surviving Patients (*n* = 129)	*p*
Serum TAC (nmol/mL)—median (*p* 25–75)	2.88 (2.29–4.00)	4.20 (3.40–5.02)	0.001
Serum melatonin (pg/mL)—median (*p* 25–75)	2.51 (2.13–3.34)	4.59 (2.73–6.89)	0.001
Serum malondialheyde (nmol/mL)—median (*p* 25–75)	3.71 (3.40–5.66)	2.96 (2.29–4.18)	0.01
Serum alpha-fetoprotein (ng/dL)—median (*p* 25–75)	11.0 (4.0–194.0)	7.0 (3.7–25.2)	0.44
Protein (g/dL)—median (*p* 25–75)	7.10 (5.80–7.65)	6.70 (6.10–7.10)	0.64
Nodules size (cm)—median (*p* 25–75)	3.5 (1.7–5.0)	3.0 (2.0–3.5)	0.41
MELD score—median (p 25–75)	15 (12–18)	15 (12–18)	0.95
Leukocytes count—median × 10^3^/mm^3^ (*p* 25–75)	4.89 (3.68–7.76)	4.75 (3.51–6.36)	0.68
Creatinine (mg/dL)—median (*p* 25–75)	1.01 (0.79–1.10)	0.90 (0.79–1.06)	0.37
BMI (kg/m2)—median (*p* 25–75)	28.53 (24.07–31.39)	27.66 (24.55–30.48)	0.53
Albumin (g/dL)—median (*p* 25–75)	3.47 (3.00–3.94)	3.32 (2.90–4.06)	0.83
Age of liver recipient (years)—median (*p* 25–75)	57 (53–63)	58 (52–62)	0.76
Age of liver donor (years)—median (*p* 25–75)	62 (50–72)	52 (36–63)	0.03
Female—*n* (%)	0	19 (14.7)	0.13
Ascites—*n* (%)	3 (18.8)	55 (42.6)	0.10
Infiltration—*n* (%)	5 (31.3)	39 (30.2)	0.99
Inside Milan criteria prior to LT—*n* (%)	15 (93.8)	124 (96.1)	0.51
Inside Milan criteria after LT—*n* (%)	11 (68.8)	107 (82.9)	0.19
Macrovascular invasion—*n* (%)	0	7 (5.4)	0.99
Microvascular invasion—*n* (%)	4 (25.0)	27 (20.9)	0.75
Multinodular tumor—*n* (%)	5 (31.3)	38 (29.5)	0.99
Portal hypertension—*n* (%)	12 (75.0)	89 (69.0)	0.78
Degree of tumor differentiation—*n* (%)			0.55
Well	12 (80.0)	95 (73.7)	
Moderate	3 (13.7)	31 (24.0)	
Poor	1 (6.3)	3 (2.3)	
Child-Pugh score—*n* (%)			0.27
A	11 (68.8)	61 (47.3)	
B	3 (18.8)	38 (29.5)	
C	2 (12.5)	30 (23.3)	
ABO blood type—*n* (%)			0.89
A	6 (37.5)	59 (45.7)	
B	2 (12.5)	10 (7.8)	
O	7 (43.8)	53 (41.1)	
AB	1 (6.3)	7 (5.4)	
Transplantation technique—*n* (%)			0.78
By-pass	6 (37.5)	42 (32.6)	
Piggy back	10 (62.5)	87 (67.4)	
Treatment prior to LT—*n* (%)	11 (68.8)	72 (55.8)	0.78
Percutaneous ethanol injection (PEI)—*n* (%)	7 (43.8)	28 (21.7)	0.07
Radiofrequency ablation (RFA)—*n* (%)	0	8 (6.2)	0.60
Transarterial chemoembolization (TACE)—*n* (%)	4 (25.0)	29 (22.5)	0.76
Liver resection—*n* (%)	0	3 (2.3)	0.99
Mixed treatment—*n* (%)	0	4 (3.1)	0.99

TAC: total antioxidant capacity; MELD: model for end-stage liver disease; BMI: body mass index.

**Table 2:** Logistic regression analysis for the variables associated with one-year liver transplantation mortality.

	Odds Ratio	95% Confidence Interval	*p*-value
Age of liver donor (age)	1.042	1.004–1.082	0.03
Serum melatonin levels (pg/mL)	0.525	0.331–0.834	0.006

## Abbreviations

AFP	alpha-fetoprotein
HCC	hepatocellular carcinoma
LT	liver transplantation
MELD	model for end-stage liver disease
